# Morphological Control of Cilia-Inspired Asymmetric Movements Using Nonlinear Soft Inflatable Actuators

**DOI:** 10.3389/frobt.2021.788067

**Published:** 2022-01-03

**Authors:** Edoardo Milana, Bert Van Raemdonck, Andrea Serrano Casla, Michael De Volder, Dominiek Reynaerts, Benjamin Gorissen

**Affiliations:** ^1^ Department of Mechanical Engineering, KU Leuven and Flanders Make, Leuven, Belgium; ^2^ Department of Engineering, Institute for Manufacturing, University of Cambridge, Cambridge, United Kingdom

**Keywords:** morphological control, embodied intelligence, nonlinear soft bending actuators, bioinspiration, artificial cilia

## Abstract

Soft robotic systems typically follow conventional control schemes, where actuators are supplied with dedicated inputs that are regulated through software. However, in recent years an alternative trend is being explored, where the control architecture can be simplified by harnessing the passive mechanical characteristics of the soft robotic system. This approach is named “morphological control”, and it can be used to decrease the number of components (tubing, valves and regulators) required by the controller. In this paper, we demonstrate morphological control of bio-inspired asymmetric motions for systems of soft bending actuators that are interconnected with passive flow restrictors. We introduce bending actuators consisting out of a cylindrical latex balloon in a flexible PVC shell. By tuning the radii of the tube and the shell, we obtain a nonlinear relation between internal pressure and volume in the actuator with a peak and valley in pressure. Because of the nonlinear characteristics of the actuators, they can be assembled in a system with a single pressure input where they bend in a discrete, preprogrammed sequence. We design and analyze two such systems inspired by the asymmetric movements of biological cilia. The first replicates the swept area of individual cilia, having a different forward and backward stroke, and the second generates a travelling wave across an array of cilia.

## Introduction

In traditional robotics, the body and the brain are two separate entities. All control is encoded in software algorithms, while the body is following the software instructions. In biological systems, however, control is distributed differently. Studies have found that in organisms control is partly outsourced from the brain to the body (Pfeifer and Bongard 2006). This is possible because the geometry and composition of the body have evolved in such a way that the physics acting on it passively realize the desired functionality. This process is called morphological control and is a form of embodied intelligence. It is especially apparent in lower-level organisms that can even function without any neural computation (Hoffmann and Müller 2014). The increasing interest in embodied intelligence in biology is matched in robotics by the development of soft robots with morphological control.

**FIGURE 1:**
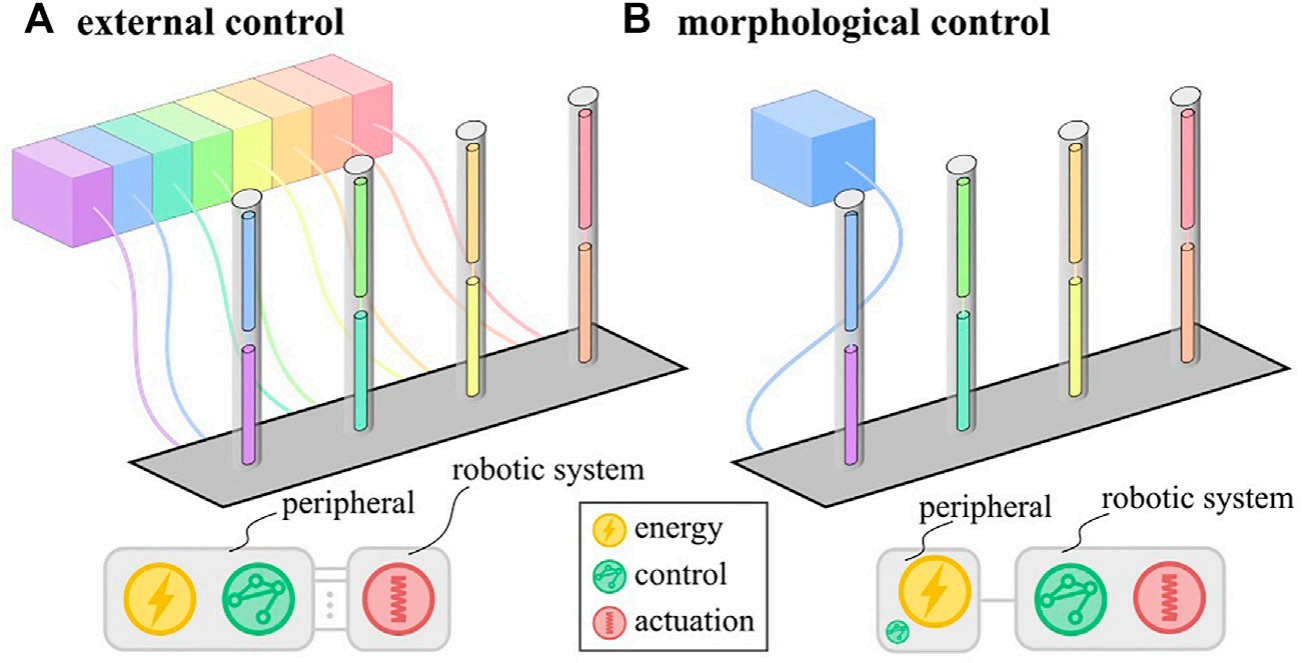
Schematic representation of external **(A)** and morphological **(B)** control schemes with distribution of the fundamental functions: energy, control and actuation. In morphological control, the control function is embodied in the robotic system.

Soft robots are made of highly compliant materials such that interactions with the environment substantially influence their deformation. This property makes them difficult to control using conventional control schemes but increases the potential for morphological control (Rus and Tolley 2015). The idea behind morphological control is to harness the physical properties of the soft system to simplify the control scheme. This simplification in control means that fewer peripheral components are needed to create a function. For soft robots with a traditional external control scheme, these components are numerous because they coordinate motion across multiple degrees of freedom algorithmically. Every actuator corresponding to such a degree of freedom needs a dedicated control loop with a pressure supply and a tether (Figure 1A). With morphological control, the coordination of motion between different actuators can be built into the physical characteristics of the system. Therefore, a single pressure supply suffices to drive the system, drastically reducing tethering (Figure 1B).

Literature presents several techniques to implement morphological control. A common approach is to use fluidic logic circuits to transform a single input signal into multiple time-varying output signals. Fluidic circuits with linear Quake-type valves have been used to make multiple tentacles oscillate in Octobot (Wehner et al., 2016) and to drive a two-state soft articulated robot devoid of electronics (Mahon et al., 2019). Similarly, fluidic circuits of soft bistable valves featuring buckling shells and kinking tubes (Rothemund et al., 2018) have been used to turn a constant input pressure into oscillating signals (Preston et al., 2019) and into controllable walking motions of a multi-state soft robot (Drotman et al., 2021).

Compared to the systems with Quake-type valves, the bistable valves have the advantage of retaining their state in case of a pressure loss and of having faster transitions between states. These properties arise from the nonlinear behavior of the valve which features snap-through instabilities. This highly nonlinear behavior is present in many soft structures either because of the hyperelastic material properties at large strains (Gent 1999) or because of geometric nonlinearities associated with large deformations (Kochmann and Bertoldi 2017). For soft fluidic actuators, this can result in relations between the internal pressure and internal volume in the actuator with a local peak and valley in pressure. The peak and valley in the pressure-volume curve (PV curve) are separated by a region that is unstable when pressure is imposed. On reaching the peak or valley pressure, the volume suddenly changes in a snap-through instability.

While these instabilities improve valve performance, they can also be directly incorporated in the actuator designs (Overvelde et al., 2015) to create soft machines that can swim (Chen et al., 2017; Arakawa et al., 2021), walk (Gorissen et al., 2019) and jump (Gorissen et al., 2020) with simplified morphological control and without need of fluidic logic circuits, further decreasing the amount of tethers.

Another form of morphological control explored in literature is to harness pressure drops over passive flow restrictors in a system of inflatable actuators for sequencing. This approach has already been implemented for the locomotion of soft robots with standard soft actuators (Futran et al., 2018; Vasios et al., 2019). Similarly, damping layers in soft actuators are also used to obtain morphological control of actuation sequences (Di Lallo et al., 2019). Combining the passive flow restrictors with snap-through actuators further discretizes the actuation sequence, yielding better locomotion (Gorissen et al., 2019) and clear sequences of state transitions in arrays of bistable elastic shells (Ben-Haim et al., 2020).

In this work, we build a system of snapping soft inflatable bending actuators inspired by biological cilia, and show that morphological control can be used to achieve the typical nonreciprocal ciliary motion patterns using a single input. Biological cilia are slender motile organelles responsible for fluid propulsion in both microorganisms and animal tissues (Jahn 1967). Due to their small size (down to 10 µm), cilia operate in low Reynolds environments, where viscous effects are predominant over inertial ones and, according to the Scallop theorem (Purcell 1977), a spatial asymmetric motion is required to propel fluid. In fact, at very low Reynolds (Re < 0.1) inertial effects of the fluid are so negligible that a simple differential speed in the effective and recovery stroke does not generate any net motion, therefore, swimming techniques like the one of the scallops (fast closing of the shell) are not effective at low Reynolds. Similar hydrodynamic conditions are experienced by flagella, cellular appendages that are used for locomotion (Bardy. 2003).

Artificial cilia (Toonder and Onck 2013) and flagella (Singleton et al., 2011) are devices that mimic biological motion in order to propel fluid at low Reynolds conditions and are becoming interesting case studies in soft robotics due to their flexible and compliant movements. Soft flagella inspired by prokaryotic bacteria have been used to propel an underwater vehicle (Armanini et al., 2021), while arrays of soft cilia are developed using magneto-elastomeric materials (Gu et al., 2020; Dong et al., 2020). Soft artificial cilia are also made using fluidic actuators and their state-of-the-art consists of a deformable pillar with two embedded cavities that are pressurized independently with two separate pressure lines (Milana et al., 2019). That means that an array of *n* cilia requires *2n* separate pressure lines and valves, which complicates the control of the device both in hardware and software. With morphological control, the aim is to recreate the sequences that form the asymmetric motions while heavily reducing the number of pressure lines, simplifying the system and enabling further scaling of the system. Conversely to other types of robotic swimmers that use snap-through phenomena to generate an impulsive thrust at high Reynolds (Mochiyama et al., 2013; Arakawa et al., 2021), in this manuscript the snap-through mechanism only serves the purpose of morphologically controlling the asymmetric motion sequence, which is the key mechanism of low Reynolds propulsion.

In the first section of this paper we introduce a design for nonlinear inflatable actuators with a bending deformation, inspired by the SliT concept (Belding et al., 2018) and characterize their nonlinear response for various geometric parameters. Further we describe the nonreciprocal movement of a single artificial cilium demonstrator, consisting of two such bending actuators stacked on top of each other and inflated with one fluidic input to create sequenced output. Finally, we describe another demonstrator with four bending actuators in an array replicating the metachronal movement in cilia arrays from a single input. With these demonstrators we realize two symmetry-breaking mechanisms found in nature (spatial and metachronal asymmetry) with one pressure input as opposed to many inputs in our previous research (Gorissen et al., 2015; Milana et al., 2020). Though the demonstrators are cm-scale prototypes, they show that morphological control can simplify peripheral hardware. This opens the way to miniaturizing systems of artificial inflatable cilia to scales relevant for applications.

## Materials and Methods

### Nonlinear Bending Actuators

As a building block to mimic ciliary movements, we designed inflatable slit-in-tube bending actuators with a nonlinear pressure-volume (PV) curve and that exhibit a snap-through instability.

The actuators consist out of an inner cylindrical latex balloon (Super-Soft Latex Rubber Tubing from McMaster-Carr) to contain the fluid and generate the nonlinearity (Overvelde et al., 2015), and an outer cylindrical shell of flexible PVC (Masterkleer PVC Clear Tubing from McMaster-Carr) to limit the strain and generate bending (Belding et al., 2018). Each end of the inner balloon is connected to the barbed end of a luer lock adapter (Plastic Quick-Turn Tube Coupling from McMaster-Carr) glued to the outer shell. The outer shell features a pattern of circumferential slits on one side. It limits the circumferential expansion of the inner latex balloon and directs its axial expansion into bending. The shape of the PV curve of the actuator depends mostly on the radii of the latex balloon and the PVC shell, while the bending deformation is guided by the slit pattern parameters. We fabricated three types of bending actuators with this design, all made of the same PVC shell (outer diameter 11.2 mm, inner diameter 8 mm, length 40 mm). Five uniformly spaced (spacing = 5 mm) slits are cut halfway the tube diameter using a craft knife. In order to prevent stress concentrations, 1 mm round perforations are made at the tips of the slits with a biopsy punch. The actuators differ in latex balloon outer diameter (6.4, 4.8 or 3.2 mm), but have the same wall thickness of 0.8 mm and length of 40 mm. The PV curves are measured by inflating the actuators with water using a custom syringe drive and registering the pressure in LabView, as shown on Supplementary Figure S4. The bending curvature is measured by capturing Movie images (Nikon 1 V3 camera) and tracking three markers along the actuator length and fitting a circle through them using the Matlab Computer Vision Toolbox. The curvature and pressure measurements are synchronized by means of an audio signal generated by the software controlling the syringe drive.

### Spatial Asymmetry Setup

A biological cilium traces a path that is different between its forward and backward stroke. The artificial recreation of this movement in literature necessitated at least two bending segments that are linked together and controlled with a phase difference between them (Sareh et al., 2012; Milana et al., 2019). Here, we fabricated and tested three types of artificial cilia each consisting of two of the previously detailed nonlinear bending actuators. One actuator sits at the base of the cilium and the other at the tip and they are connected by screwing together the luer locks at their ends. In two of the three tested composite cilia, no obstruction is present in the interconnecting luer lock so the bending actuators experience the same pressure. In the third composite cilium, the luer lock channel is restricted by a silicone plug with a needle of diameter 2 mm and length 10 mm punched through it to form a restriction (see SI for more details). The cilia are inflated and deflated with a proportional valve (Festo MPPES-3-1) and the deformations are recorded with a camera. The amount of spatial difference between forward and backward stroke is calculated by tracking the tip of the actuator and measuring the area it encloses when going through one actuation period.

### Metachrony Setup

In biological cilia arrays, every individual cilium describes a similar trajectory at the same frequency. However, there is a phase difference between cilia that are placed next to each other, creating an effective mechanism for fluid flow (Khaderi, den Toonder, and Onck 2012). To replicate this metachronal wave, we placed four nonlinear bending actuators next to each other in a row with 40 mm between each actuator. The actuators are all identical with a latex balloon outer diameter of 4.8 mm. They are connected serially to a pressure supply (Festo MPPES-3-1) with narrow tubes that act as flow restrictors (inner diameter 0.2 mm, length 0.5 m). The deformation of the individual actuators is registered using the three-point tracking technique detailed before in the section on the nonlinear bending actuators.

### Simulations

The response of the two experimental setups is compared to numerical simulations of the equivalent lumped fluidic systems. Considering an interconnected system of actuators and flow restrictors, the system is simulated using the following equations, where *p*_*i*_ and *∆v*_*i*_ are the pressure and the volume change of the *ith* actuator, while *∆p*_*i*_ and *q*_*i*_ are the pressure drop and the flow across the *ith* restrictor that connects actuator *i-1* and *i* together.

$$\begin{array}{c} \Delta p_{i}\left(q_{i}\right)+p_{i}\left(\text{Δ}v_{i}\right)-p_{i-1}\left(\text{Δ}v_{i-1}\right)=0 \end{array}$$ (1)

$$\begin{array}{c} \frac{d\text{Δ}v_{i}}{dt}=q_{i}-q_{i+1\;} \end{array}$$ (2)

Those equations were already adopted in previous works to simulate the morphological control of interconnected inflatable actuators in (Glozman et al., 2010; Di Lallo et al., 2019; Gorissen et al., 2019; Vasios et al., 2019; Ben-Haim et al., 2020).

The system of equations is solved numerically to find the evolution of the volume in each actuator over time. The relations for *p*_*i*_ *(∆v*_*i*_ *)* are implemented as interpolations of the experimentally measured PV curves of the actuators. The pressure’s drop across the restrictors *∆p*_*i*_ *(q*_*i*_ *)* is modeled using the Darcy-Weisbach law for a cylindrical geometry where the flow of air is approximated as incompressible for simplification:

$$\begin{array}{c} \Delta p_{i}=\mathrm{sgn}\left(q_{i}\right)\frac{8\rho f_{d}}{{\text{π}}^{2}}\frac{L_{i}}{d_{i}^{5}}q_{i}^{2} \end{array}$$ (3)

With *L*_*i*_ the length of the restrictor and *d*_*i*_ its inner diameter. As a simplification, the fluid density *ρ* = 1.78 kg/m³ and the Darcy friction factor *f*_*d*_ = 0.04 are assumed to be constant in this paper. In reality, however, $\rho \left(p_{i}\right)$ and $f_{d}\left(q_{i}\right)$ are functions of the local pressure and flow rate, respectively. For the systems presented in this paper, the flow regime is assumed laminar so then $f_{d}=64/Re$ and Eq. (3) reduces to

$$\begin{array}{c} \Delta p_{i}=\mathrm{sgn}\left(q_{i}\right)\frac{128}{\pi }\mu \frac{L_{i}}{d_{i}^{4}}q_{i} \end{array}$$ (4)

With *µ* the dynamic viscosity of the fluid in the tube. This equation shows that scaling all dimensions of the tube by a factor α scales $\text{Δ}p_{i}$ for the same $q_{i}$ with $\alpha^{-3}$ . The required fluid flow $\left(q_{i}\right)$ to achieve a fixed normalized actuator velocity scales with $\alpha^{3}$ itself. Therefore, the relation between $\text{Δ}p_{i}$ and the normalized actuator kinematics is independent of the system size. So even though all experiments and simulations presented in this paper are on the cm-scale, similar systems at the mm or µm-scale exhibit the same sequencing dynamics given the same pressure signal.

For low flows or wide and short tubing (no flow restrictors), the pressure drop becomes negligible (*∆*p_i_(q_i_) *= 0*), and Equation (1) reduces to:

$$\begin{array}{c} p_{i}\left(\text{Δ}v_{i}\right)-p_{i-1}\left(\text{Δ}v_{i-1}\right)=0 \end{array}$$ (5)

The system then behaves quasi-statically and the model is identical to the one reported by Overvelde et al. (Overvelde et al., 2015). At any point in time, the pressure in each actuator is then equal to the pressure imposed by the source. The actuator volumes follow by interpolating the actuator PV curves at that pressure. In case multiple equilibrium volumes exist for the same pressure, the volume closest to the one in the previous time step is selected. This approach is used for the analysis of the spatial asymmetry setups without flow restrictors.

## Results and Discussion

### Nonlinear Actuators Characteristic

**FIGURE 2:**
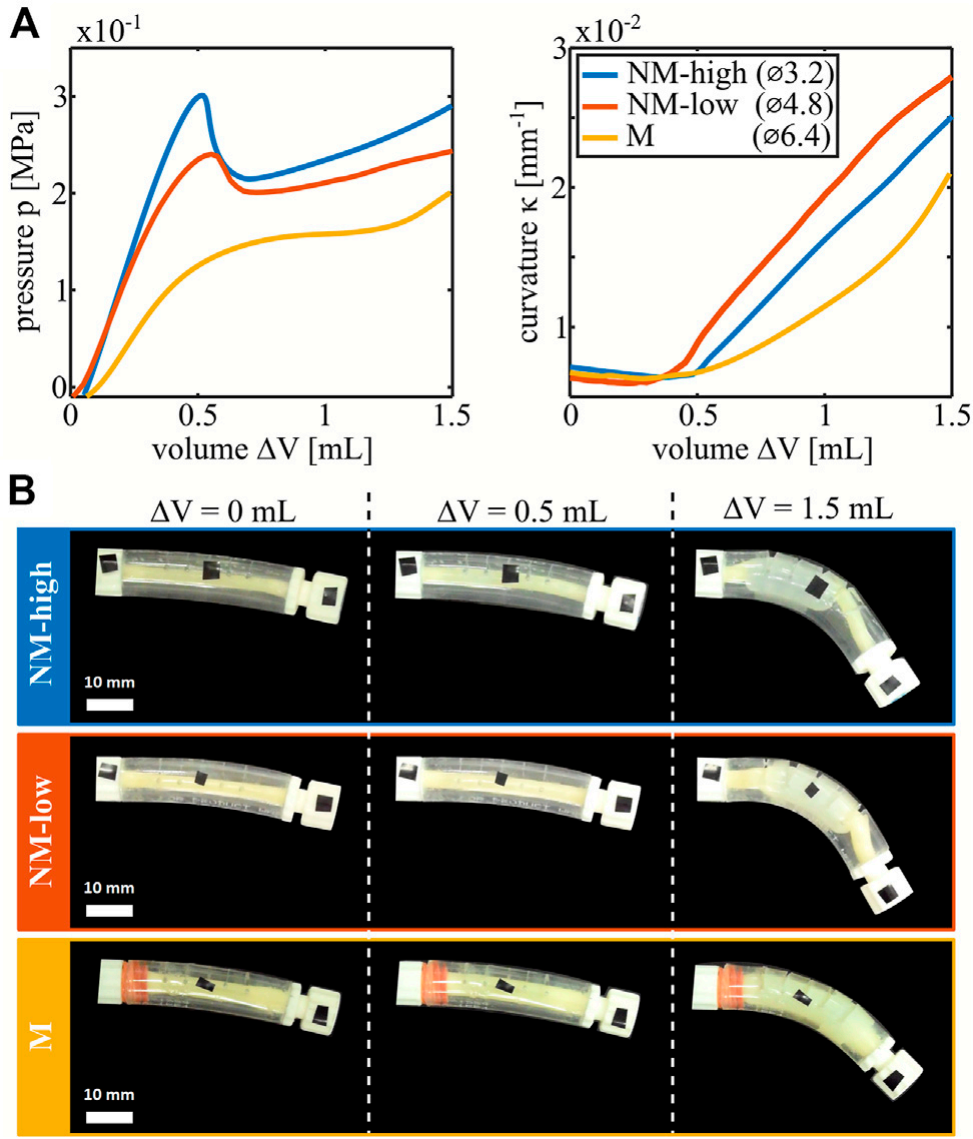
Characterization of the three types of manufactured nonlinear bending actuators differentiated by the outer diameter of the latex balloon. **(A)** PV curves and curvature-volume curves of the actuators. **(B)** Deformed configuration for each type of actuators at 0, 0.5 and 1.5 ml of volume increase.

The PV and curvature-volume curves (KV curves) of the three nonlinear bending actuators described in the Materials and Methods section are plotted in Figure 2A, with snapshots of the deformations shown in Figure 2B. The shape of each PV curve is determined by the interactions between the inner latex balloon and outer PVC shell. When inflated without constraints, a cylindrical latex balloon deforms uniformly until a local aneurysm rapidly develops, creating a peak in the PV curve. Upon further inflation, the aneurysm grows at nearly constant pressure (Supplementary Figure S1).

In the manufactured nonlinear bending actuators however, the outer shell constrains the maximum radial expansion of the latex balloon. As soon as the outer shell and the latex balloon make contact, the balloon effectively stiffens, and the slope of the PV curve increases. For small initial radii of the latex balloons, contact occurs shortly after the aneurysm formation with associated peak such that a valley is created after the peak in the PV curve. For larger initial radii of the latex balloon, the events of aneurysm formation and contact approach each other until the corresponding peak and valley coincide and disappear. This results in decreasing nonlinearity between the actuators with a latex balloon outer diameter of 3.2 mm (which we label as being highly non-monotonic, NM-high), 4.8 mm (low nonmonotonic, NM-low) and 6.4 mm (monotonic, M). For the non-monotonic actuators, a snap-through event occurs when pressure is controlled and passes their peak or valley which leads to a rapid change in volume at constant pressure. The KV curves in Figure 2A are nonlinear as well. The reason is that the bending moment is proportional to the product of the internal pressure and the cross-sectional area of the tube (Gorissen et al., 2013). Initially, cross-sectional area is low, so the bending moment is negligible. For monotonic actuators, the latex tube area and internal pressure gradually grow so the curvature slowly ramps up. For the non-monotonic actuators, as discussed previously, an aneurysm with increased area rapidly forms on passing the peak in pressure, leading to a kink in the KV curve. Due to this change in cross-sectional area, different states with the same pressure in the nonmonotonic actuators have different corresponding curvatures. In the non-monotonic actuators, snap-through is therefore associated with a large change in curvature.

### Spatial Asymmetry

**FIGURE 3:**
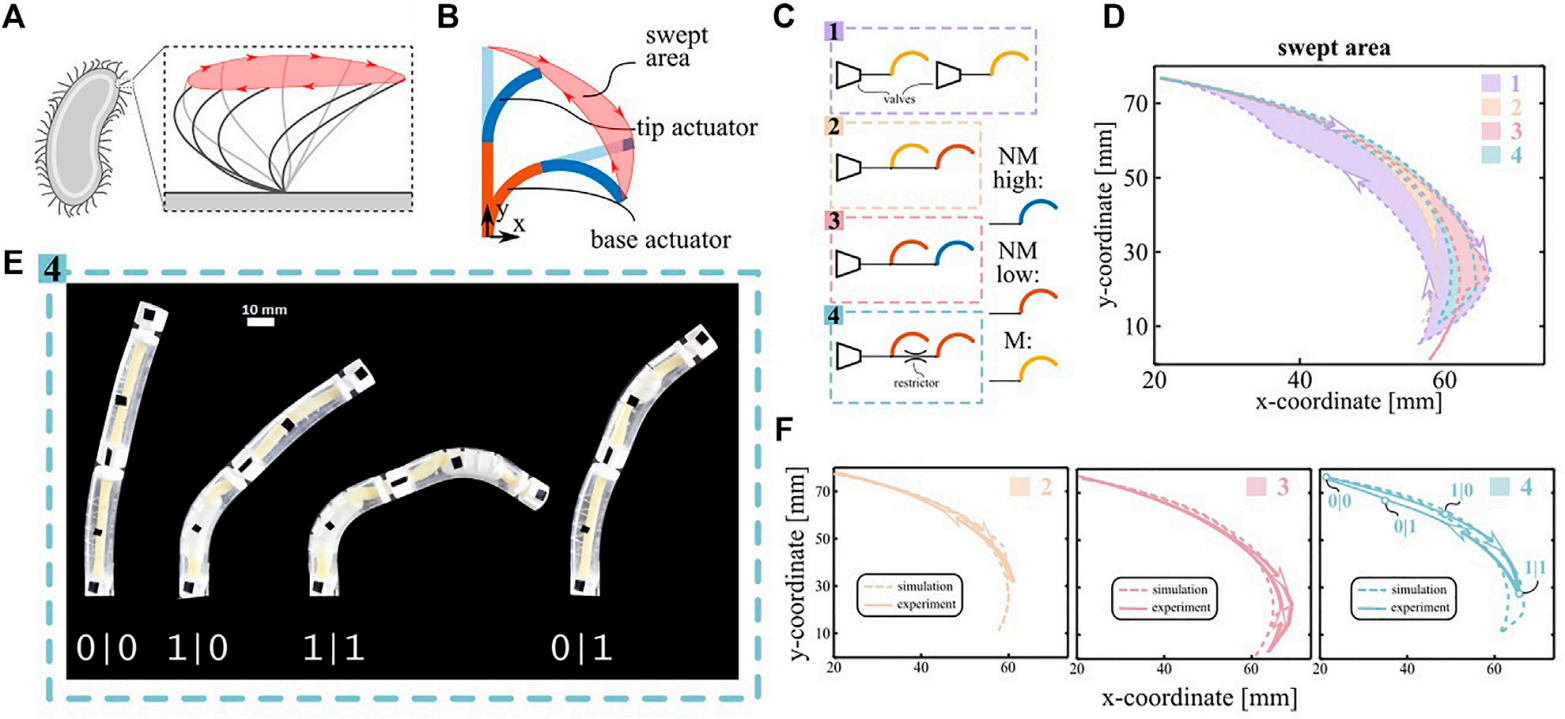
Spatial asymmetry demonstrator. **(A)** Sketch of the stroke motion of a biological cilium. The swept area is highlighted in red. **(B)** Sketch of a two-segment artificial cilium. Two actuators are stacked on top of each other. The nonreciprocal sequence of the two segments generates a swept area. **(C)** Schematic of the equivalent fluidic model for the four configurations described in the main text. **(D)** Cilium trajectories predicted by the simulations for the four configuration. **(E)** Snapshot of the actuation pattern of configuration 4. The nonreciprocal motion can be deduced by the asymmetric snapping sequence of the actuators (0 pre-snapping, 1 post-snapping). **(F)** Comparison between simulated and experimental swept area for the morphologically controlled cilia. The four states depicted in subfigure E are reported in the plot of configuration 4.

Locomotion at low Reynolds numbers relies on nonreciprocal motion (Purcell 1977), which is characterized by the cilia following a different path between forward and backward stroke (Figure 3A). Therefore, we created a simple artificial cilium by serially connecting two nonlinear bending actuator segments (Figure 3B) and tethering them to a pressure source.

We designed four such configurations, characterized them experimentally and simulated them using the experimental PV and KV curves of the individual segments (Figure 2A) as inputs. Configuration 1 on Figure 3C features two separately controlled M-type actuators and no morphological control mechanisms. This configuration is externally controlled to obtain the maximal swept area achievable and serves as a baseline. Configurations two and three exploit morphological control generated by nonlinearities during quasi-static inflation with a common pressure source ramping up and down between 0 and 0.3 MPa at a rate of 5 kPa/s. Configuration two consists of a M actuator (base) paired with a NM-low actuator (tip), and configuration three consists of a NM-low (base) paired with a NM-high (tip). Finally, configuration four exploits dynamic pressure’s drops over flow restrictors between two NM-low segments for morphological motion control. To amplify the pressure’s drop, the input pressure signal was made more dynamic by inflating to 0.3 MPa in 1 s, staying there for 12 s and ramping back to 0 MPa at a rate of 60 kPa/s.

Fig. 3D compares the simulated area swept by the tip of the different artificial cilia systems, which is a measure of the flow they can generate at low Reynolds (Milana et al., 2020). The simulated areas of the configurations with morphological control are repeated in Figure 3 and compared to experimental results (Supplementary Movie S1, S2 and S3) which are in good agreement. Configuration 1, in which no morphological control is implemented, generates a swept area of 282 mm^2^ and serves as a reference for the other areas (100%). For the quasi-static configurations, the simulated swept areas are 9% (configuration 2) and 18% (configuration 3). The latter swept area is higher because configuration three features a higher number of snap-through instabilities (four compared to two), increasing the asymmetry between inflation and deflation. Compared to the quasi-static tests, the dynamic pressure drops in configuration four generate a larger swept area of 29%. The reason is that the pressure drop generates a higher asymmetry between the appearance and disappearance of the aneurysms in the two segments than possible by tuning the geometry of the actuators. Moreover, the swept area in configuration four could be increased even further by using a tighter flow restrictor or a more dynamic input signal. Indeed, additional simulations for varying sizes of the flow restrictors show that the swept area can potentially increase up to 80% for two equal NM-low actuators (Supplementary Figure S5). In order to assess the contribution of the bistability of NM-low actuators over the flow restrictor effect, we simulated the swept area of 2 M actuators (monostable) interconnected with the same varying size of flow restrictor, where the results are also plotted in Supplementary Figure S5. We observe that the NM actuators would generally lead to higher swept areas. The difference is particularly prominent for larger sizes of flow restrictors, which correspond to lower pressure drops. In fact, if the actuators have the same snapping points, even a small pressure drop will cause a phase-shift between the two snap-through instabilities, and, therefore, a symmetry breakage with a quasi-discrete sequence. Conversely, if the actuators are monotonic, a small pressure drop induces a small time-delay with a continuous sequence that does not significantly break the spatial symmetry.

Anyway, the swept area generated by any of the morphologically controlled cilia remains small compared to the externally controlled reference. Indeed, whereas the forward stroke follows the same path in all cases, the morphologically controlled systems do not track the reference backward stroke closely. This behavior can be explained by the curvature-pressure curve of the NM-low actuator (Supplementary Figure S6). On inflating the actuator with a pressure source, it snaps from the peak to the other point on the curve with the same pressure, which represents a large change in curvature on the curvature-pressure curve. On deflation, the actuator snaps from the valley to the other point with the same pressure, which represents a small change in curvature on the curve. Therefore, the snapping instability on deflation plays a small role in discretizing the response for both the dynamic as the quasi-static case, which makes morphological control less effective. However, this limitation is not intrinsic to the morphological control but is due to the particular design of the used nonlinear bending actuators.

Knowing the kinematics of the cilia it is possible to get an estimate of the net fluid flow speed per cycle that can be potentially generated in a fluid channel at low Reynolds. At very low Reynolds, the swept area has an efficiency of roughly 0.5 (Khaderi et al., 2010), which means that the amount of net fluid motion on the beating plane in one cycle is half of the swept area. The net fluid flow speed, therefore, depends on the channel height and on the beating cycle frequency. Assuming a channel height of 100 mm and considering the beat time of 18 s used in the experimental characterization, the swept area of configuration four is expecting to pump fluid at low Reynolds at a net flow speed of about 1.4 mm/min. When normalized to the cilia length this value is very low compared to other inflatable artificial cilia (Milana et al., 2019), because of the smaller swept area as discussed above, and, mainly, due to the very slow actuation cycle (18 s), which was chosen this long in the experiment to simplify the characterization.

### Metachronal Wave Demonstrator

**FIGURE 4:**
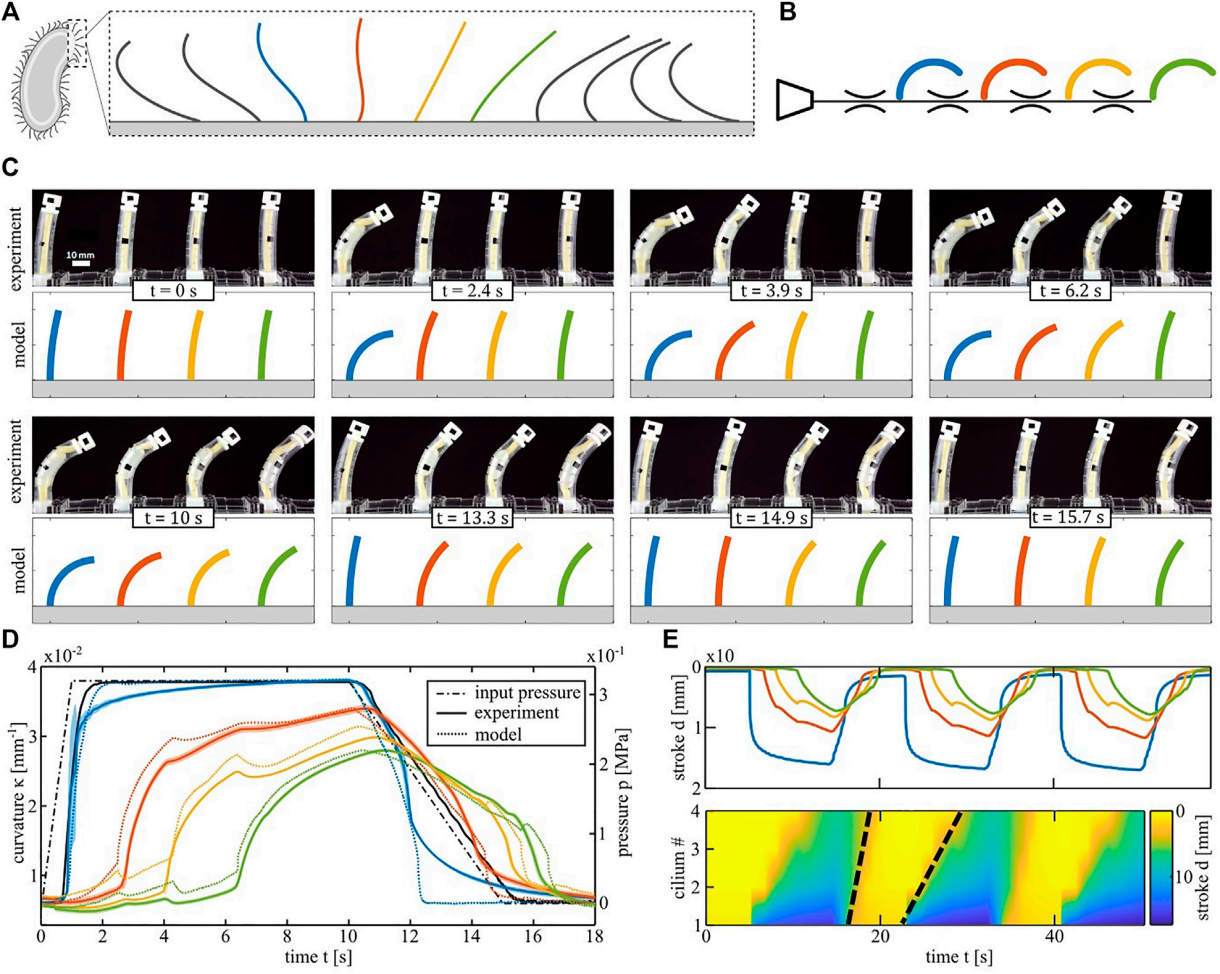
Metachronal wave demonstrator. **(A)** Sketch of the metachronal wave in a biological cilia array. **(B)** Schematic of the equivalent fluidic model of the artificial cilia array. The color code is different from the one of the spatial asymmetry test. Here the four actuators are the same type NMl. **(C)** Artificial cilia array configuration at different instants of a cycle. Snapshots from the experiment are compared with simulated curvatures of the equivalent model. **(D)** Curvature response of the four cilia during one period following the pressure input (black line). Experimental results are averaged over five cycles. The mean is plotted in solid lines and the coloured areas represent the standard deviation in pressure. Dotted lines are the output of the fluidic circuit analysis. **(E)** Spatiotemporal graphs (kymographs) of the tip vertical displacement of the four cilia. Zero displacement is depicted in yellow and the maximum in blue. The Black dashed lines track the wavefront of the metachronal wave and their slope represents the wave speed.

Another symmetry breaking principle in arrays of biological cilia is metachrony, where a phase difference is present between the motions of neighboring cilia (Figure 4A). We mimic this wave-like progression in a system of four identical nonlinear bending actuators connected to a single pressure input, as schematically shown on Figure 4B. If the actuators are connected to the input without any flow restrictors, all actuators bend at the same time (Supplementary Movie S4). However, if flow restrictors are placed between the actuators, the system exhibits a time delay between actuators in response to a trapezoidal pressure input. This is depicted in the snapshots of Figure 4C, with the experimental set-up at the top and the equivalent simulation results at the bottom. Figure 4D plots the pressure input signal and the curvature of each actuator in a cycle. The solid lines are the averages over five measurement cycles and the dotted lines are the simulated curvatures. In order to directly visualize the emergence of a metachronal-like wave at the array level, we plot the vertical displacement d of the actuator tips in the spatiotemporal graph (kymograph) in Figure 4E. Yellow fields correspond to the initial position of each actuator and the blue end of the color scale corresponds to actuators in a bent position. The two black dashed lines are an approximate visual indication of the wave fronts on inflation and deflation and their slopes represent the travelling speed. The wave speed upon inflation (23 mm/s) is 57% slower than upon deflation (53 mm/s) even though the slope of the pressure input on deflation was made less steep to equalize the wave speeds. The average phase shifts between the cilia calculated from those wave speeds (Guo et al., 2014) are 0.19 π rad for inflation and 0.08 π rad for deflation. The explanation for this difference between inflation and deflation is twofold. The first reason is that the slope of the PV curve of each actuator after snapping on inflation is less steep than the slope after snapping on deflation. Since a fluidic actuator is equivalent to an electrical capacitance from a network perspective, this slope is inversely proportional to the time constant of an RC circuit. This means that on inflation, the pressure in each actuator recovers more slowly from disturbances such as the dynamic pressure drop when an actuator snaps. Once an actuator snaps on inflation, it takes more time for its pressure to reach the same level as at the instant before the snap. Since the pressure in the first actuator acts as a source for the next actuators, the latter takes longer to reach their snapping points on inflation, resulting in a slower wave speed than on deflation.

The second reason is that the difference between the peak pressures of the actuators and the top plateau of the pressure input signal (0.32 MPa) is lower than the difference between the actuator valley pressures and the bottom plateau of the input signal (0 MPa). Therefore, in the region where the actuator pressures are near the peaks and valleys, lower flows are generated when the input pressure signal is high than when it is low. The second effect reinforces the first so during inflation it takes more time to overcome slight differences between different actuator peaks and to recover from fluctuations in pressure caused by the snapping. This is visible on Supplementary Figure S7, where the simulated pressure inside every actuator during a cycle is shown, and on Supplementary Movie S6, with an animation of where each actuator is on its PV curve in function of time.

It is noticeable the cilia strokes decrease along the chain, because of a reduction in perceived pressure. However, very recent work pointed out that instabilities can also be used to alleviate that problem (Vasios et al., 2021), where two-way transition waves were created in a tube that is not restricted in length. The combination of both technologies (transition waves and cilia with instabilities) can thus be seen as an avenue for elongating the cilia chain.

## Conclusion

In conclusion, we have shown how cilia-inspired motion sequences can emerge in systems of snapping inflatable bending actuators connected to a single pressure source by harnessing the morphological properties of the system itself.

We have designed and realized two systems that display morphological control to mimic ciliary deformations, either on an individual cilium level to create spatial asymmetry or on a group level to create metachronal waves. In contrast to previous studies on inflatable cilia, utilizing morphological control showed that the same functionality can be created while drastically reducing the number of pneumatic tethers. Moreover, the results we achieve on the cm-scale generalize to similar systems at smaller scales. This is particularly important as one of the main applications of artificial cilia arrays are foreseen to be pumps and mixers for microfluidics as well as microswimmers, which all require orchestrated movements at the microscale. In the general context of fluidic actuation, as it is infeasible for artificial cilia in large arrays to be individually addressed with tubes and valves, group actuation techniques using morphological control are paramount in creating such miniaturized systems.

We foresee that further research in both the design and manufacturing of miniaturized bending actuators with tuned peak-valley PV curves opens broad perspectives towards inflatable microsystems with advanced functionality.

## Data Availability

The raw data supporting the conclusions of this article will be made available by the authors, without undue reservation.
