# Supplementary material for: Morphological Control of Cilia-Inspired Asymmetric Movements Using Nonlinear Soft Inflatable Actuators
Source: Front Robot AI. 2022 Jan 3;8:788067. doi: 10.3389/frobt.2021.788067 (PMC8762291; doi:10.3389/frobt.2021.788067)
Supplement: Supplementary file 6 [file DataSheet1.docx]

Supplementary Material

# Nonlinear bending actuators design and principles

The nonlinear bending actuator is composed of a latex balloon placed inside a flexible PVC shell, with lateral slits to obtain a differential stiffness. As simplified model to explain the deformation, it is convenient to consider the equivalent Young's modulus of the PVC tube and latex balloon in contact Eb and to assume the side with the slits as a bulk material with a Young's modulus Es < Eb (Fig. S2). When the latex tube is pressurized and gets in contact with the outer PVC shell, the actuator bends as results of this differential stiffness. Indeed, the axial strains ε_z_ are asymmetric across the section of the shell and, as consequence, a curvature forms. From Euler-Bernoulli beam theory the following is known:

$$\begin{aligned} M_{x}=\int_{A} \sigma_{z}\left( y-y_{n} \right)\text{d}A\#1 \end{aligned}$$

where M_x_ is the bending moment around the x-axis, A the cross-section area, σ_z_=E ε_z_ the axial stress and y_n_ the y-coordinate of the neutral axis of the section. Therefore, bending deformation depends only on the axial stresses. According to Muller [1], the equation of pressurized vessels can be used to determine the axial stress in inflated tubes. Given the tube diameter D with a thickness t at a pressure p, it follows that:

$$\begin{aligned} \sigma_{z}=\frac{pD}{4t}\#2 \end{aligned}$$

When the latex tube is in contact with the flexible PVC, it behaves as a pressurized vessel and equation 2 holds. For non-monotonic actuator this occurs when the latex tube snaps (p=p*) and the aneurysm forms. In this way the pressure-volume nonlinear behavior of the latex can be harnessed to generate a snapping response of the actuator, which is paramount to obtain a sequence with morphological control. If the inner tube has a diameter large enough to contact the shell before reaching p*, the actuator has a monotonic behavior.

# Nonlinear bending actuators fabrication steps

The actuators are assembled out of the following components pictured in (figure S3B): a flexible PVC tube (Masterkleer PVC Clear Tubing, from McMaster-Carr), a latex tube (Super-Soft Latex Rubber Tubing Semi-Clear, from McMaster-Carr) and two luer-lock couplings (Plastic Quick-Turn Tube Coupling Nylon, from McMaster-Carr). The size of the luer-lock couplings has to fit the latex tube inner diameter.

As first step, a stencil (Fig. S3A) with the slits dimensions is printed on printer paper, cut out, covered in glue and wrapped around the PVC tube. To make the structure stiffer against the forces of cutting the slits, a metal rod matching the inner diameter of the PVC tube is inserted into it. Subsequently, slits are cut in the tube with a craft knife following the features of the paper stencil. The holes at the slit corners are cut using a 1 mm biopsy punch. The excess material is removed as well as the metal rod.

Then, the latex tube is cut at the same length as the PVC tube (40 mm) and inserted in the latter. Before the assembly of the luer-lock couplings, a temporary mark is made on the outside showing where the screw thread of the connector begins. Subsequently the couplings are inserted in both sides of the latex tube such that their marks are aligned. When the external ring of the couplings is glued to the PVC tube with cyanoacrylate adhesive (super glue), the marks are aligned with the leftmost corners of the slits. This makes sure that the bending planes of the nonlinear bending actuators align when they are screwed together. The advantage of the luer-lock coupling is that the actuators can be very easily connected together. On the other hand, the luer-lock becomes a rigid component in the soft actuators, decreasing the overall deformability of the structure. A sketch of the final actuator is depicted in Fig. S3C.

# PV curve test setup

The syringe pump consists of a 10 mL syringe connected to a linear stage driven by a stepper motor (Fig. S4). The entire system is filled with an incompressible fluid (water) from a tank and any air that might remain in the system after filling it is extracted with an additional syringe. That way, the volume change in the actuator is known directly from the displacement of the syringe plunger imposed by the stepper motor in open loop. The pressure is measured by a sensor (PR-21S pressure transducer, Keller) placed along the tube connecting the actuator to the syringe pump. A LabVIEW program synchronizes the measured pressure values with the imposed displaced volume values to construct the PV curve of the actuator under test. The deformation of the actuator is captured by a camera (Nikon 1 V3) and synchronized to the PV measurements through a sound signal from the LabVIEW code. Three square markers are cut from black electrical tape and attached to the actuator. They allow a motion tracking algorithm in MATLAB to determine the average curvature of the actuator from the video feed. The flow rate induced by the syringe pump is kept small (6 µL/s) to assure quasi-static conditions.

# Reference

1. Müller I, Strehlow P, Marder M. Rubber and Rubber Balloons: Paradigms of Thermodynamics. Physics Today, 2005, 58, 55.

# Supplementary Figures and Tables

## Supplementary Figures


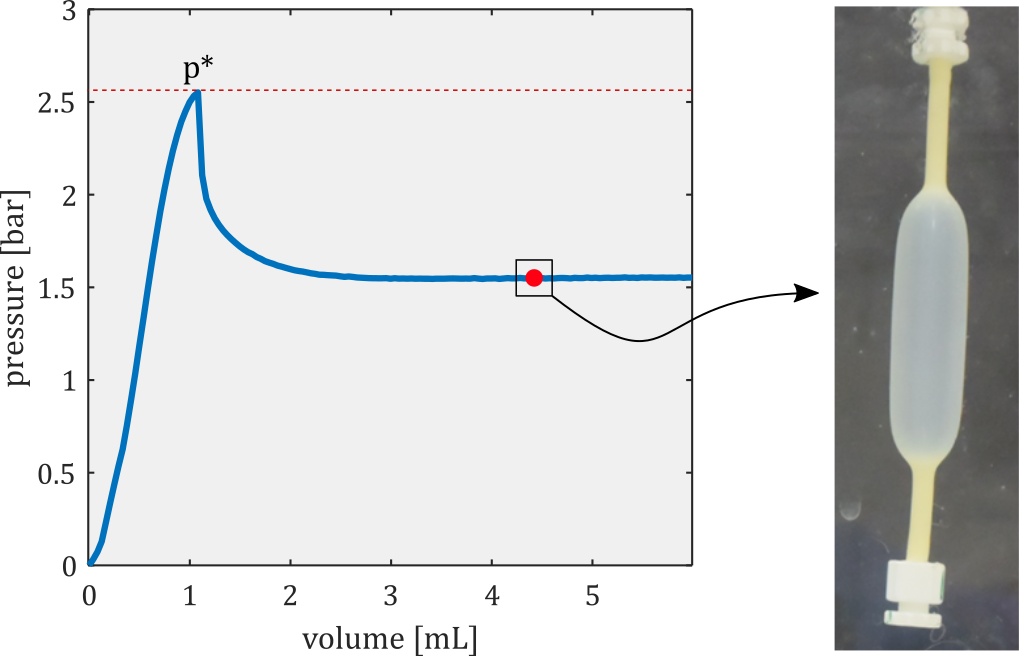


**Supplementary Figure 1.**. Pressure-Volume measurement of an unconstrained cylindrical latex balloon. After reaching critical pressure peak p*, an aneurysm forms that inflates at constant pressure.


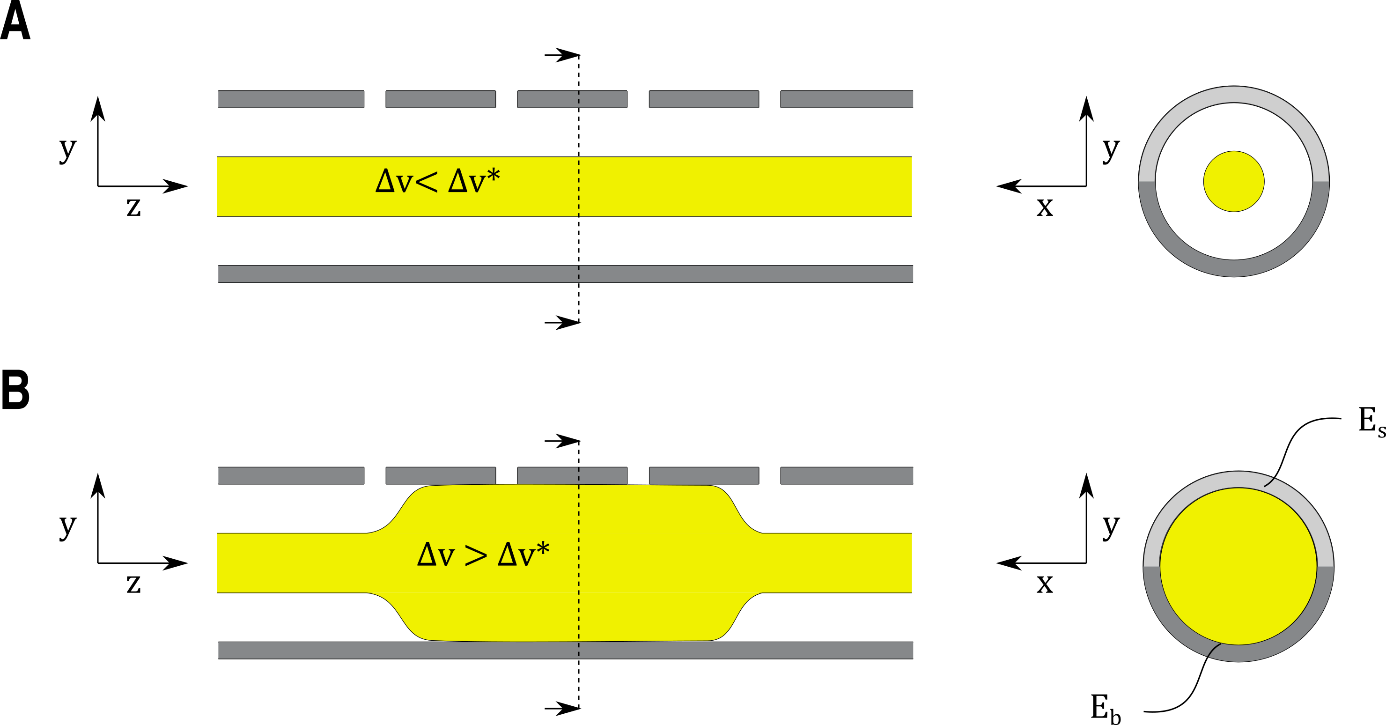


**Supplementary Figure 2.** Sketch of the longitudinal and cross-sectional view of the nonlinear bending actuator. A. Volume in the latex balloon is below the critical value ∆v* (where p(∆v*) = p*) while in B volume is above the critical value. Es and Eb are the equivalent Young modulus of the slits and bulk side of the actuator, where Es<Eb.


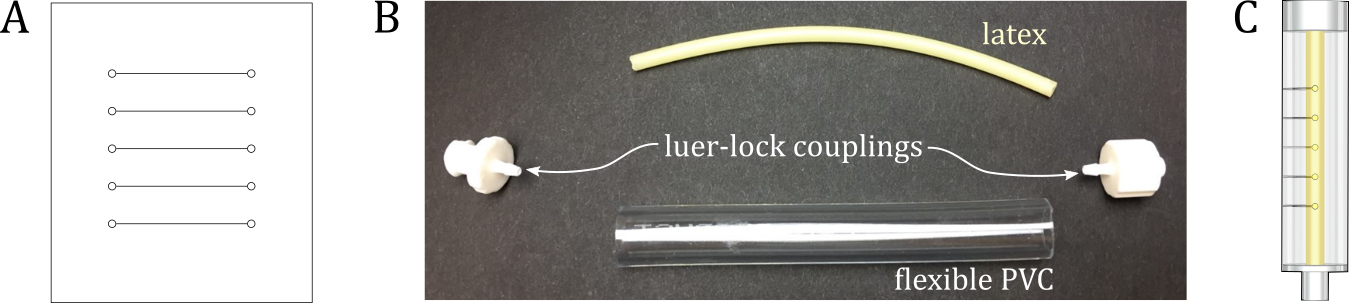


**Supplementary Figure 3.** A. Slits stencil for the cutting step. B. Three components of the actuator: latex balloon (inflatable part), flexible PVC tube (outer shell) and luer-lock couplings. C. 3D sketch of the assembled actuator.


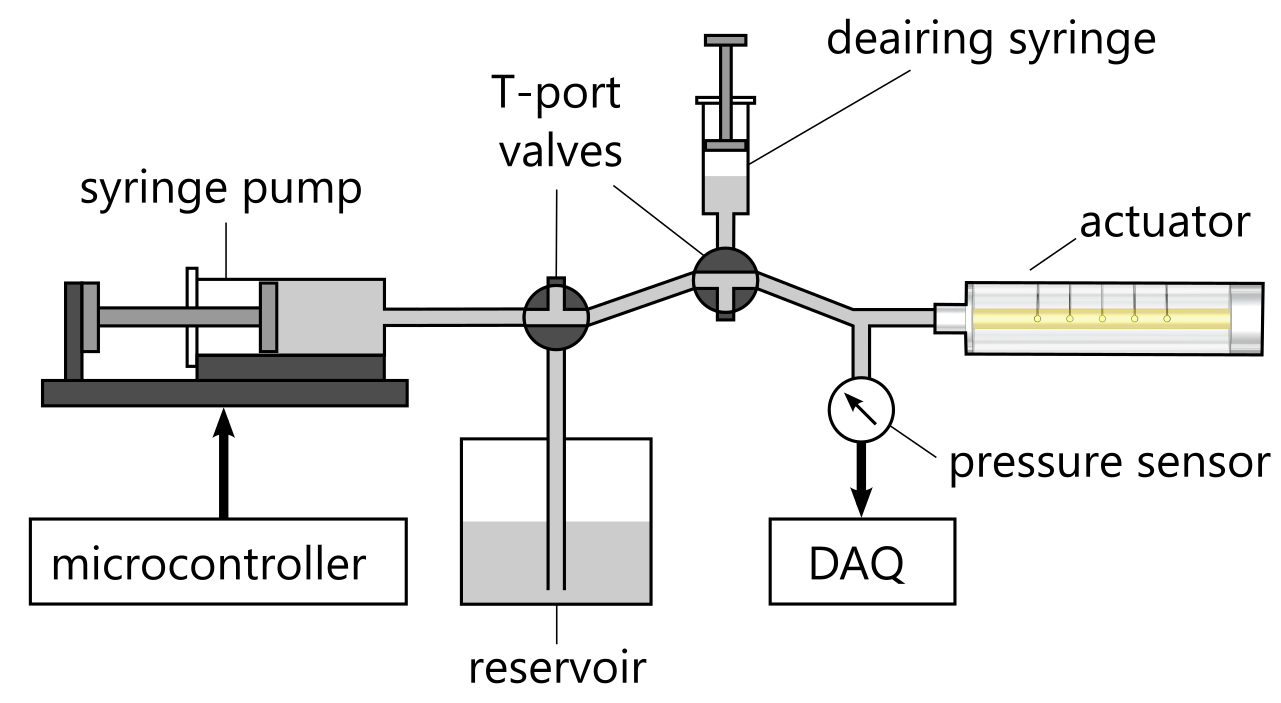


**Supplementary Figure 4**. Schematic of the experimental setup to measure the pressure-volume curve of the actuators.


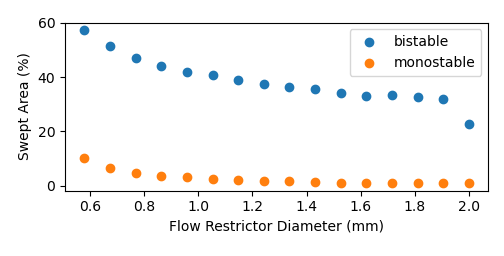


**Supplementary Figure 5.** Comparison between simulated swept areas generated by two NM-low (bistable) actuators and two M (monostable) actuators interconnected with a varying size of the diameter of the flow restrictor.


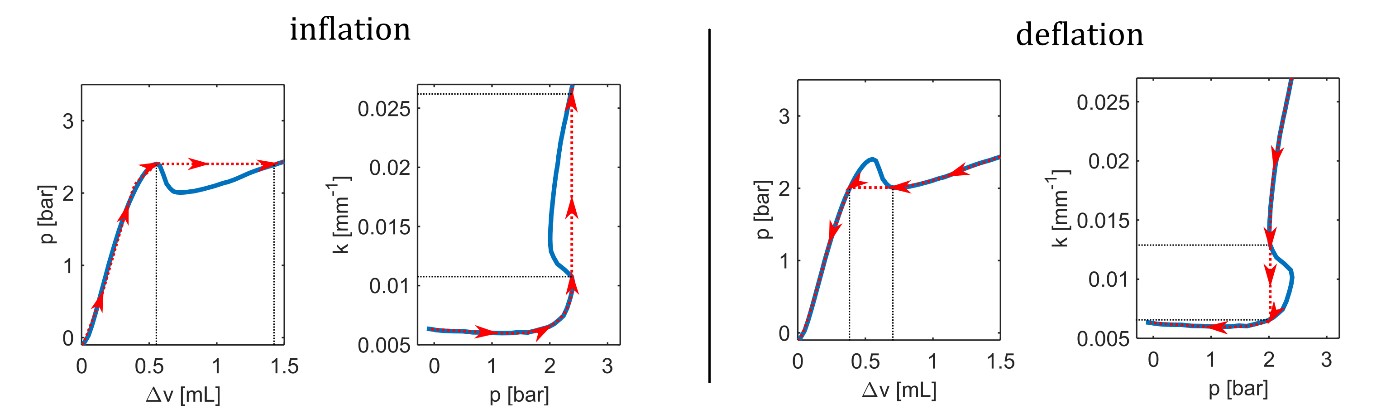


**Supplementary Figure 6.** Different path along the PV curve of NM-low actuator for inflation and deflation. Due to the different slopes after snapping on inflation and deflation, the volume exchange and, therefore, curvature change, is much higher on snap-through on inflation than on deflation. This phenomenon limits the asymmetry in the overall nonreciprocal motion.

**
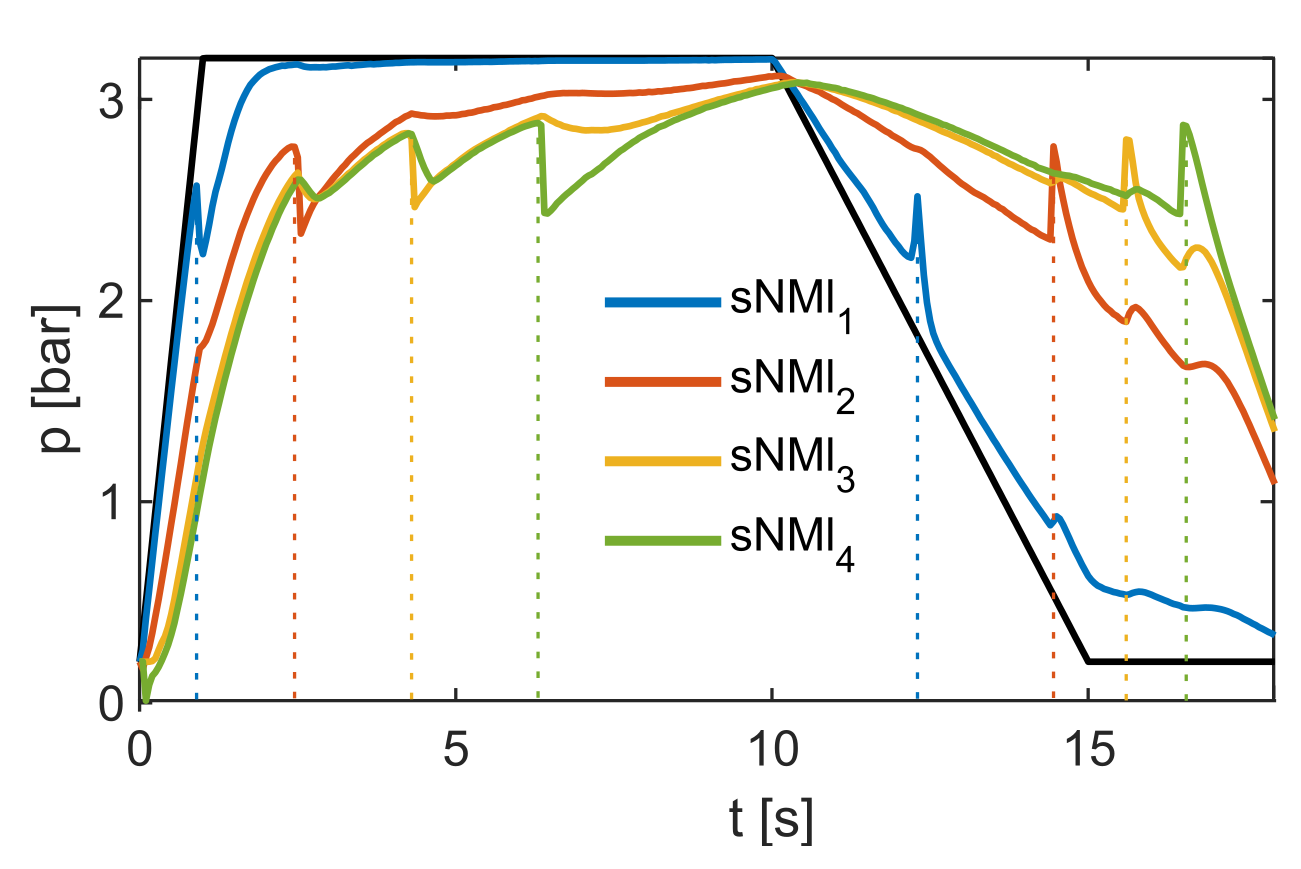
**

**Supplementary Figure 7**. Simulated pressure variation during one cycle for the four cilia. Response delay during deflation progressively reduces, diminishing the metachronal shift.

## Supplementary movies legends

**Movie S1.** Spatial asymmetry test: sM+sNMl (quasi-static). This movie shows the quasi-static asymmetric behaviour of a cilia-like bi-segment nonlinear actuator.

**Movie S2.** Spatial asymmetry test: sNMl+sNMlh (quasi-static). This movie shows the quasi-static asymmetric behaviour of a cilia-like bi-segment nonlinear actuator.

**Movie S3.** Spatial asymmetry test: sNMl1+sNMl2 (dynamic). This movie shows the dynamic asymmetric behaviour of a cilia-like bi-segment nonlinear actuator.

**Movie S4.** Metachrony test: no restrictors. This movie shows the response of four equal nonlinear bending segment connected to the same pressure source without any flow restrictor in series.

**Movie S5.** Metachrony test: restrictors. This movie shows the response of four equal nonlinear bending segment connected to the same pressure source with flow restrictors in series. The pressure drop across the restrictors induced a phase difference, resembling ciliary metachrony.

**Movie S6.** Metachrony test: simulation output of the pressure-curvatures variation of the four actuators during a cycle. This movie shows the simultaneous variation of the pressure-curvature curves in the simulation of the metachrony setup.
